# The Oxygen Mixture, a New Anæsthetic Combination

**Published:** 1868-11

**Authors:** E. Andrews

**Affiliations:** Prof. of Principles and Practice of Surgery, Chicago Medical College


ARTICLE XLVI.

### THE OXYGEN MIXTURE, A NEW ANÆSTHETIC COMBINATION.

By E. ANDREWS, M.D., Prof. of Principles and Practice of Surgery, Chicago Medical College.

Every surgeon who has seen the prompt and pleasant anæsthetic action of the nitrous oxide gas, so much used by dentists, has wished that in some way it might be made available in general surgery. The patient usually goes under the influence in 30 or 40 seconds, and wakes with equal promptness, without vomiting or other unpleasant symptoms, all of which is in striking contrast with the slowness, the nausea, and the discomforts of chloroform and ether. There have been, however, great obstacles to the use of the gas, owing to its evanescent action. The oxygen contained in it is in a state of chemical combination, so that it is not available for oxygenation of the blood; hence if any attempt is made to continue its action, the patient becomes purple in the face, showing all the signs of asphyxia; subsultus tendinum then supervenes, and shortly after he almost ceases to breathe, and, if allowed nothing but pure nitrous oxide, would doubtless die in a few minutes.

I have for some time been experimenting, to see whether by the addition of free oxygen to the nitrous oxide, a mixture would not be obtained, by which a patient might be anæsthetized for an indefinite period without danger of asphyxia, and thus render gas available for the most prolonged operations of surgery. These experiments are not yet finished, but they have advanced far enough to show that the preparation, which I have named the Oxygen Mixture, is certainly available for a large part of our operations, and that for pleasantness, and probable safety, it is infinitely superior to chloroform, ether, or unmixed nitrous oxide. The following facts and experiments show the present state of our knowledge on the subject:—

In the first place, pure nitrous oxide, when given for brief operations, appears to be the safest anæsthetic known. Chloroform, in American and European hospitals, kills one out of about every 3600 patients who take it; but the Colton Dental Association, a company with branches in all our principal cities, established for the sole purpose of extracting teeth, has on its books over sixty thousand cases of anæsthesia by nitrous oxide, without a single death caused by the anæsthetic.

Now, it cannot be supposed that the addition of a moderate amount of free oxygen, in mechanical mixture, to nitrous oxide can produce any new danger; on the contrary, by removing all possibility of asphyxia, it must be eminently an element of safety.

To test this question, the following experiments were performed:—

EXP. 1. A large rat was placed in a glass jar on a perforated floor, beneath which was a stratum of lime-water to absorb the carbonic acid produced by its breathing. To make more sure of this result, a jet of lime-water spray was thrown into the jar at frequent intervals during the experiment. I then turned on a small stream of pure nitrous oxide gas, which, being fifty per cent. heavier than atmospheric air, settled to the bottom, and expelled the atmospheric air by displacement. In two minutes the animal fell over upon its side, breathing slowly with deep-labored inspirations. The respirations continued to become slower, until, at the end of ten minutes, they ceased entirely, and life was found to be extinct. The death was doubtless from asphyxia.

EXP. 2. Another rat was placed in the jar under the same conditions, and exposed to an oxygen mixture consisting of about one-fourth of free oxygen to three-fourths of nitrous oxide. In two and a half minutes he was so completely anæsthetized that he could not be made to respond to pinching or pushing. There was no panting, or laboring for breath, as when pure nitrous oxide was used, but the respiration was rather slow, and very gentle. He was kept in the mixture half an hour, and then removed, still perfectly anæsthetized. In five minutes he began efforts at walking, and in ten seemed to be perfectly restored to his natural condition.

Exp. 3. A rat was placed in the jar and given the oxygen mixture, containing 25 per cent. pure oxygen. This being more than is contained in the atmosphere, diluted the nitrous oxide too much, which, together with the fact that the animal was less susceptible than the former, prevented full anæsthesia. He fell into a sort of intoxicated condition, without appearing to be fully unconscious, and continued thus throughout the experiment. At the end of 30 minutes the gas was shut off, and the animal shortly recovered his sobriety.

Exp. 4. The same animal was again exposed to the oxygen mixture for half an hour, with precisely the same results as before.

Exp. 5. To test the relative safety of the oxygen mixture as compared with ether, my friend Dr. Sherman took the same rat, after his recovery from experiment No. 4, and dropped into the jar a little sulphuric ether. In a short time he was unconscious, and in two minutes was dead.

Exp. 6. A lady had an anchylosed knee, to which I wished to restore motion by forcible flexion. Having a dread of ether and chloroform, she inhaled the oxygen mixture in the proportion of one-third free oxygen to two-thirds nitrous oxide. In forty seconds she was perfectly anæsthetized, without any blueness of the countenance, or laboring for breath. There was a little pallor about the lips. I broke up the adhesions of the joint by flexing and extending it forcibly. She probably inhaled the gas about two minutes, felt no pain, and awaked without nausea.

Exp. 7. A young woman took in my presence the mixture as prepared by Dr. Rogers, dentist, for the extraction of a tooth. There was, as before, a slight pallor of the prolabia, but no asphyxiated purpling of the face. The tooth was extracted without pain, and the patient awoke without nausea.

Exp. 8. A woman, aged 42, had anchylosis of the right hip, with contraction of the flexors of both knees, fixing those joints at a right angle. I desired to cut all the hamstring tendons of both limbs, and to break up by force the adhesions of the anchylosed hip. The gas was given from a 30-gallon elastic bag, with an imperfect inhaler. The mixture contained one-third free oxygen. Owing to the imperfection of the inhaler, it was found impossible to prevent the patient getting considerable atmospheric air with the gas, so that the anæsthesia was less perfect, and slower than in the former instance. After inhaling it for nine minutes, she became unconscious, and I severed all the hamstrings. I then endeavored to break the adhesions of the head of the femur, but found they were too firm, and I desisted. The operations lasted about three minutes, when she was allowed to recover, which she did without nausea, though she had a meal in the stomach. Twice during the inhalation there was a sort of pallor of the face, with very faint duskiness, which induced me to suspend the administration of the gas a few respirations.

EXP. 9. Mrs. R. had ingrowing, painful nails on both feet. Ten months ago she took ether for the extraction of one of them. She was of a very nervous temperament, was slow in coming under the influence of the ether, and after partially awaking remained delirious, and distressed a considerable time. Three months afterwards she took pure nitrous oxide for the extraction of a tooth. She was anæsthetized in about one minute, and felt no pain, but the countenance was blue with asphyxia, and she was delirious a good while after waking. She felt uncomfortable for several days. Six months afterwards she was again anæsthetized by Dr. Reber, a dentist, who had prepared the oxygen mixture at the suggestion of Dr. Sherman. The gas contained one-third free oxygen. She was anæsthetized in one and three-quarter minutes, and in that condition Dr. Sherman split the offending toe-nail and tore out the proper half of it without causing any pain. She inhaled the gas for three minutes in all. On awaking, she was as usual delirious, which state, however, continued only fifteen minutes, a much shorter time than after ether or pure nitrous oxide. There was no blueness nor pallor of the lips during inhalation, and on her waking she was much more comfortable than after anæsthesia with the other articles.

Dr. Reber has given the oxygen mixture to several patients for the extraction of teeth, and states that it uniformly acts more agreeably than unmixed nitrous oxide.

Dr. Rogers, a dentist of this city, states that he has used a mixture containing one-third free oxygen for several years, and that in his opinion it is far pleasanter than unmixed nitrous oxide.

Some months ago some such mixture was proposed in England, but was overthrown, I think, by the influence of Dr. Richardson, who argued, on theoretical grounds merely, that it would not be successful, nor safe. I cannot learn that it was ever actually tried in Europe.

Prof. Watt, of the Dental College in Cincinnati, has been experimenting, I understand, on what involves partly the same principle. I am informed that he gives alternately inspirations of nitrous oxide and atmospheric air, and thus both avoids the asphyxia, and is able to continue the inhalation a long time. I have written to him inquiring about his results, but have received no answer.

The above experiments are by no means sufficient to settle the value of the oxygen mixture, but they give strong reason to think that it will prove the safest, and by far the pleasantest, anæsthetic known. As to its safety, it is highly significant, that a rat which had been twice immersed in the mixture for half an hour without injury, was killed in two minutes by ether; and yet ether is far safer than chloroform.

It is my impression that the best proportion of oxygen will be found to be one-fifth by volume, which is the same as in the atmospheric air. There are some points requiring care in the management, in order to insure success. As the oxygen dilutes the nitrous oxide, it is necessary to be very careful to exclude all atmospheric air, or else the anæsthesia will be imperfect. The inhaler must be taken into the mouth, the lips very carefully closed around it, and the nares compressed by the person administering the anæsthetic. For the same reason, great care should be taken to secure purity of the gases, otherwise the mixture will be too weak to control some patients. I have found, by introducing phosphorus into a bell glass of what was supposed to be very pure nitrous oxide, that it contained considerable free oxygen, which doubtless was from included atmospheric air; and therefore four times the bulk of free, inert nitrogen must have been present also, to weaken the power of the article.

The oxygen is best prepared by taking pure chlorate of potash mixed with a little black oxide of manganese, and placing them in a copper retort and applying heat. The gas should pass through four washing-bottles, just as the nitrous oxide does. The same bottles will answer. As the nitrous oxide is fifty per cent. heavier than oxygen, it is better to pass it into the gasometer first. The oxygen coming afterwards, passes up through it, and hastens the mixing. It is better to let them stand a day or two, if possible, before using, to complete the mixture, but this is not essential.

Dr. Evans, the well-known American dentist in Paris, asserts that the ordinary nitrous oxide is very far from pure, even when well made. He states that he has been in the habit of purifying his gas by mechanically condensing it to a liquid under high pressure. This liquid, being absolutely pure nitrous oxide, is then allowed to reassume the gaseous form in a bag, or a gasometer. He finds that gas thus purified, only requires about half the usual quantity to anæsthetize a patient.

It seems probable, therefore, that the oxygen mixture will enable us to anæsthetize a patient for the longest as well as for the shortest surgical operations, and that it is safer and pleasanter than any anæsthetic known. There are, however, some inconveniences about it, on account of its great bulk. For office use, and also in hospitals, this is no objection, as it can be kept in a gasometer; but for outside patients it can only be carried in a large rubber bag. In city practice, among the higher classes, however, this is no obstacle, as the bag can always be taken in a carriage, without attracting observation.

I shall continue my experiments, and report the results at a future time.

